# Incremental Load Respiratory Muscle Training Improves Respiratory Muscle Strength and Pulmonary Function in Children with Bronchiectasis

**DOI:** 10.1155/2024/8884030

**Published:** 2024-05-23

**Authors:** Xiaolong Chen, Shidong Hu, Xiaohui Jia, Bingbing Zeng

**Affiliations:** ^1^Rehabilitation Medicine Center, The Second Affiliated Hospital of Wenzhou Medical University, Wenzhou 325027, Zhejiang, China; ^2^Intelligent Rehabilitation Research Center, China-USA Institute for Acupuncture and Rehabilitation, Wenzhou Medical University, Wenzhou 325027, Zhejiang, China; ^3^Department of Pediatric Respiratory, The Second Affiliated Hospital and Yuying Children's Hospital of Wenzhou Medical University, Wenzhou 325027, China; ^4^Center of Traditional Chinese Medicine, The Second Affiliated Hospital of Wenzhou Medical University, Wenzhou 325027, Zhejiang, China

## Abstract

**Methods:**

Participants underwent respiratory muscle training for 24 weeks. The main results were changes in respiratory muscle strength and pulmonary function indices (forced expiratory volume in 1 s (FEV1), forced vital capacity (FVC), FEV1/FVC, peak expiratory flow rate (PEF), forced expiratory flow 25–75% (FEF25–75%), and maximal midexpiratory flow 75/25 (MMEF75/25)) before, 12 weeks after, and 24 weeks after the intervention. The secondary outcomes were changes in the exercise load and work rate, exercise work, Leicester Cough Questionnaire (LCQ) scale, and Fatigue Severity Scale (FSS).

**Results:**

Compared with before the intervention, after 24 weeks of respiratory muscle training, the maximal inspiratory pressure (MIP) and maximal expiratory pressure (MEP) were significantly enhanced (*P* < 0.05), while FVC, FEV1, and PEF were significantly increased (*P* < 0.01). FEF25–75 and MMEF75/25 values showed significant improvement compared to those before training (*P* < 0.05). The exercise loading, work, and exercise work rate of expiratory muscle training were significantly improved compared to those before intervention (*P* < 0.05). The LCQ score increased significantly (*P* <  0.001), and the FSS score decreased significantly (*P* <  0.001).

**Conclusion:**

Incremental load respiratory muscle training effectively improved children's lung function over the long term, improved the strength of their inspiratory and expiratory muscles, and improved their quality of life.

## 1. Introduction

Bronchiectasis is a chronic lung disease. The main clinical manifestations are cough and expectoration, a large amount of airway secretions, and restricted movement [1]. The incidence rate of bronchiectasis is increasing yearly with the ageing of the population [2]. With the development of the medical detection technology, the number of cases of bronchiectasis in children is also increasing. Patients with bronchiectasis usually have varying degrees of respiratory muscle weakness [3]. Inspiratory muscle weakness leads to slower contraction of inspiratory muscles and insufficient lung ventilation, which cannot provide adequate inspiration thus affecting expiration [4]. Expiratory muscle weakness leads to cough weakness and repeated production of sputum, which leads to difficulty in sputum clearance and finally causes bronchial infection [5, 6]. Conventional drugs such as bronchodilators and anti-inflammatory drugs cannot continuously improve symptoms, and long-term use of corticosteroids can actually lead to respiratory muscle dysfunction [7]. Bronchiectasis can have harmful effects on lung function and even lead to permanent structural loss of the small airways [8]. The repeated deterioration of the disease will have a harmful impact on the lung function and growth of children and seriously affect their quality of life [9].

Respiratory muscle training is a lung rehabilitation program [10], and other programs include active cycle of breathing techniques (ACBT), postural drainage, chest expansion training, and other airway clearance techniques [11]. Studies have shown that the respiratory muscle training can improve the strength of the inspiratory and expiratory muscles in critically ill patients, and it has a certain tolerance level [12]. Respiratory muscle training can not only promote sputum discharge in the short term and maintain airway cleanliness but also improve the lung function, enhance respiratory muscle strength and endurance, and improve quality of life [13].

Previous studies have shown that respiratory muscle training can improve the lung function and respiratory muscle strength in various respiratory diseases to varying degrees [14]. For example, in patients with chronic obstructive pulmonary disease (COPD), improvements in respiratory muscle strength and endurance can be observed after systemic or specific respiratory muscle endurance training [15–17]. Respiratory muscle training, as a type of lung rehabilitation program, is gradually being applied to the treatment of patients with bronchiectasis [18]. However, there is limited research on the therapeutic effect of respiratory muscle training on children with bronchiectasis, and there is also a lack of research on the long-term therapeutic effect of therapy in children with bronchiectasis.

Therefore, the purpose of this study was to explore whether respiratory muscle training could effectively improve the lung function of children with bronchiectasis in the long term. We hypothesized that respiratory muscle training would have a significant effect on improving respiratory muscle strength and lung function in children with bronchiectasis.

## 2. Methods

### 2.1. Study Design

This study was a 24-week pre- and post-controlled clinical trial to train respiratory muscles in children with bronchiectasis. This study was conducted under the guidance of Chen Xiaolong's cardiopulmonary physiotherapist. It was approved by the Ethics Committee of the Second Affiliated Hospital of Wenzhou Medical University (2022-k-31-01). All patients signed informed consent forms.

### 2.2. Study Population

From June 2021 to December 2021, seventeen children with clinically stable bronchiectasis aged 5–15 years were included. The inclusion criteria included children, who were in stable condition (no deterioration in the previous month), had sputum production and could not be discharged within one year before receiving treatment, and had never received any lung rehabilitation plan, with bronchiectasis confirmed by chest CT images in the Second Affiliated Hospital of Wenzhou Medical University. The exclusion criteria included children with cystic fibrosis, deterioration of the disease in the past month, participation in the lung rehabilitation program, inability to implement the lung rehabilitation program or inability to participate in medical treatment, and any physical therapy contraindications.

### 2.3. Study Procedures

Children were trained with a Xeek threshold loading device. First, the threshold loading device was used to detect the patient's lung function, measure MIP and MEP, and then set 30% of the MIP and MEP as the initial training intensity according to the assessment results. The assessment process is as follows: before performing respiratory muscle training in children with bronchitis, there is a section for evaluating respiratory muscle function in the data feedback system connected to the respiratory muscle trainer. The child underwent eight forceful inhalation and exhalation sessions to obtain evaluation data (MIP and MEP), and then set training thresholds based on the evaluation results for training. The training intensity continued and gradually increased to the maximum allowable intensity. The training time was 10 times/session, 8 sessions a day, with 4 sessions of inspiratory muscle training and 4 sessions of expiratory muscle training. Training was performed 5 days a week, and the whole training cycle lasted for 24 weeks. During the whole study period, the children's training was completed under the guidance of the therapist, and the children maintained the routine use of respiratory drugs.

### 2.4. Assessments

The severity of bronchiectasis was evaluated with the BSI score [19] before respiratory muscle training. The respiratory muscle strength and lung function of children were evaluated before respiratory muscle training, after 12 weeks of training and after 24 weeks of training. The main lung function indicators were FVC, FEV1, FEV1/FVC, PEF, FEF25–75, and MMEF75/25. The LCQ rating scale was used to measure the quality of life [20]. The degree of fatigue in children was evaluated with the FSS [21]. At the same time, respiratory trainers recorded the changes in muscle strength, exercise loading, exercise work rate, exercise work, and other related indicators.

### 2.5. Statistical Analysis

The measured data were analysed and categorized into primary and secondary results. The normality of the data was evaluated. The normal distribution of data was evaluated using one-way ANOVA with repeated measures to assess the changes in variables before and after the group. If the data were not normally distributed, the Mann‒Whitney test was used. Each time point and the changes compared to the baseline were compared. A *P* value <0.05 was considered to indicate significance.

## 3. Results

**Figure 1:**
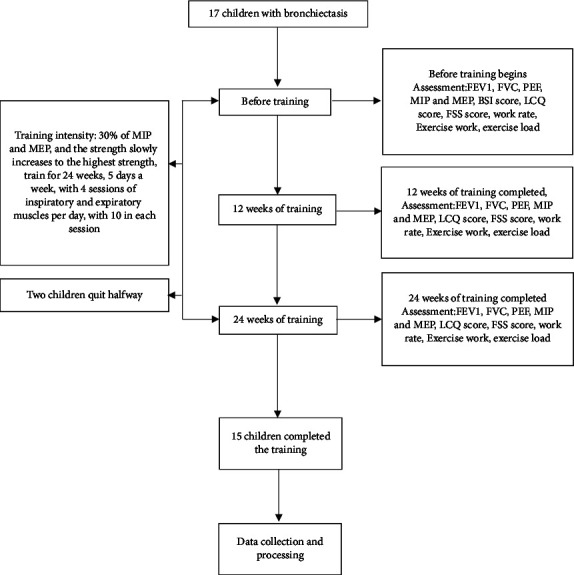
Study flowchart (intention-to-treat analysis).

Seventeen children with clinically stable bronchiectasis participated in this study. Two of them were unable to use the device correctly and interrupted the study (see Figure 1).

### 3.1. Demographics and Anthropometrics

The subjects were similar in age, sex, height, and weight before and after treatment; the severity of bronchiectasis was assessed by the BSI score (see Table 1).

### 3.2. Respiratory Muscle Strength and Pulmonary Function Indices

Before training, the respiratory muscle strength of the children with bronchiectasis significantly weakened, and the PEF significantly decreased. After 12 weeks of the respiratory muscle training, the expiratory muscle strength (MEP) of the children improved compared to before training, but the improvement was not significant (MEP: *P* = 0.071) (see Table 2), and the PEF value was also significantly higher than before training (*P* = 0.001) (see Table 3). The FEV1 and FVC of the children were significantly improved (FEV1: *P* = 0.004; FVC: *P* = 0.011) (see Table 3). After 24 weeks of training, the strength of the expiratory and inspiratory muscles of the children were significantly increased compared to before training (MEP: *P* = 0.02; MIP: *P* = 0.035) (see Tables 2 and 4). The patient's tolerance for the expiratory exercise loading, the expiratory work rate, and the expiratory work also significantly increased (expiratory exercise loading: *P* = 0.023; expiratory work rate: *P* = 0.007;expiratory work: *P* = 0.04) (see Table 2). However, throughout the 24-week inspiratory muscle training cycle, there was no significant increase in children's tolerance to the inspiratory exercise loading, the inspiratory work, and the inspiratory work rate (inspiratory exercise loading: *P* = 0.11; inspiratory work rate: *P* = 0.147; inspiratory work: *P* = 0.426) (see Table 4). The main indices of the lung function (FVC, FEV1, and PEF) in the children also showed significant improvement (FVC: *P* = 0.001; FEV1: *P* = 0.0004; PEF: *P* = 0.0001) see Table 3. FEV1/FVC showed little change throughout the training cycle (*P* = 0.173) see Table 3. The small airway index indicated that children with bronchiectasis had obvious small airway dysfunction. After 24 weeks of training, FEF25–75 and MMEF75/25 increased significantly (FEF25: *P* = 0.0001; FEF50: *P* = 0.001; FEF75: *P* = 0.045; MMEF75/25: *P* = 0.012) (see Table 3).

### 3.3. FSS and LCQ Scale

Before training, the children were prone to fatigue and their qualities of life decreases. After 24 weeks of training, compared with those before training, the fatigue level of the children with bronchiectasis decreased, the FSS score significantly decreased (*P*=0.0003), the LCQ score significantly increased (*P*=0.0001), and the quality of life significantly improved (see Table 3).

### 3.4. Effect Size

The effect size of expiratory and inspiratory muscle-related indicators is shown in Tables 2 and 4, respectively. The effect size of lung function-related indicators is shown in Table 3.

## 4. Discussion

According to the results of this study, high-intensity respiratory muscle training significantly and effectively improved the lung function, respiratory muscle strength, and quality of life of children with bronchiectasis.

Previous studies have shown respiratory muscle weakness in patients with bronchiectasis [22, 23]. The average expiratory muscle strength in children with bronchiectasis in this study was 63.28 cm H_2_O, while the average inspiratory muscle strength was 59.37 cm H_2_O. The root cause is not yet clear, but the possible cause is functional weakness related to excessive inhalation [24]. Inflammation, excessive expansion, and airway obstruction can lead to impaired diaphragmatic function. During exercise, excessive inflation can lead to a sharp increase in the inspiratory threshold load, increase the respiratory work, and the ability of the inspiratory muscles to generate pressure also decreases [25–27], inhaling muscle weakness can significantly affect autonomous breathing [28]. The decrease in expiratory muscle strength can impair the effectiveness of coughing, reduce the expiratory flow rate, and reduce the discharge of respiratory secretions [29]. Previous studies have shown that respiratory muscle training can effectively improve the strength of the inspiratory and expiratory muscles [30, 31]. The results of this study showed that after 24 weeks of respiratory muscle training, the respiratory muscle strength of children was significantly increased compared to that before, with an increase of 21.05 cm H_2_O (35%) in inspiratory muscle strength and 27.59 cm H_2_O (44%) in expiratory muscle strength, and the peak expiratory flow rate was significantly increased as well, and the PEF value increased by 1.04 L/s (26%). Previously, studies have shown that adult patients with bronchiectasis, after 8 weeks of inspiratory muscle training, experience a significant increase in inspiratory muscle strength by 21.4 cm H_2_O [32]. The inspiratory muscles are both skeletal in shape and function, so when appropriate physiological loads are applied, their response to training is similar to that of any skeletal muscle [33]. After continuous training, the diaphragmatic muscle strength can be continuously enhanced [34, 35], generating greater strength driven by the respiratory muscles. An increase in expiratory muscle strength increases the expiratory flow rate, improves the cough ability, promotes the outwards movement of sputum, and reduces the adhesion of sputum [36]. The enhancement of the expiratory muscle strength can better activate the patient's cough force [37].

Bronchiectasis severely damages the lung function [38]. Previous studies have shown that respiratory muscle training can improve FEV1 and FVC in COPD patients [39] and significantly improve FVC and total lung capacity (TLC) in CF patients with a training intensity of 80% MIP value [40], and COPD patients can generate greater inspiratory volume and faster respiratory flow rate after respiratory muscle training [41]. However, there is limited research on bronchiectasis. Before the start of this study, the lung function of the children was tested, and it was found that the main indicators of the lung function, such as FVC and FEV1, decreased to varying degrees. The average FEV1 was 1.72 L, and the average FVC was 2.31 L. Bronchiectasis can have varying degrees of small airway function involvement, with FEF25% of 3.08 L, FEF 50% of 1.55 L, FEF 75% of 0.59 L, and MMEF 75/25 of 1.27 L. Moreover, the recovery of the small airway function is more difficult [42]. After 24 weeks of respiratory muscle training, the children's FVC and FEV1 significantly improved (FEV1 increased by 0.36 L (21%), FVC increased by 0.49 L (21%)), and their small airway function also improved significantly (FEF25 increased by 0.9 L (29%), FEF50% increased by 0.42L (27%), FEF75% increased by 0.36 L (61%), and MMEF75/25 increased by 0.44 L (35%)). Previous studies have shown that inspiratory muscle training can improve the lung function and respiratory muscle strength in children with bronchiectasis, and the results show significant improvement [43] in the lung function and inspiratory muscle strength in children with bronchiectasis after 8 weeks of inspiratory muscle training. However, this study only focuses on inspiratory muscle training, which has certain limitations.

Ventilation restriction may lead to a decrease in exercise endurance and physical activity in patients with bronchiectasis. Patients with bronchiectasis may experience significant symptoms, such as exercise intolerance and fatigue [32], which have varying degrees of impact on their quality of life. Respiratory muscle training can improve exercise tolerance and prevent the progression of respiratory muscle fatigue [44]. Before and after training, the fatigue level of the children was scored using the FSS scale. Measure quality of life using the Leicester Cough Questionnaire (LCQ). The score ranges from 3 to 21 points, and the higher the score is, the smaller the impact of cough on quality of life. The questionnaire consists of three aspects: physical, psychological, and social [45]. The research results indicate that after respiratory muscle training, the FSS score decreased by 22.58 (43%) and the LCQ score increased by 6.84 (68%), indicating a significant improvement in fatigue and quality of life for the affected children. Previous studies have shown that after 3 months of chest physical therapy, the LCQ of patients with bronchiectasis has significantly improved, increasing by 1.3 [46].

The respiratory muscle training used this time was threshold incremental load training, which is one of the most widely used training methods. Children with bronchiectasis are highly adaptable to this training method and have no other side effects. Moreover, the trainer is connected to the device and can provide visual feedback, providing relevant respiratory information during respiratory muscle training. The training method can be presented in the form of games, improving the compliance and interest of children and facilitating data storage. In addition, the trainer is easy to carry and is also suitable for home training. This study was aimed to establish a long-term investigation to facilitate better long-term tracking of patient treatment outcomes. The research results indicate that respiratory muscle training can be included in the treatment plan for children with bronchiectasis. The advantage of threshold increasing load training is that it is relatively easy to adapt to gradually increasing resistance, higher pressure loads can be better tolerated, and the training effect is also significant. Previous studies have shown that increasing load training can increase inspiratory muscle strength by 44% [22].

This study also has certain limitations, such as the lack of a control group, the inclusion of few cases, and the fact that younger children may not be suitable for this plan. Further research will continue in the future, designing more comprehensive randomized controlled trials with a larger number of cases. In addition, in-depth research should be conducted on the mechanism of respiratory muscle training in children with bronchiectasis, such as respiratory mechanics. We made full use of instruments such as diaphragmatic ultrasound and diaphragmatic electromyography to more accurately study the changes in the diaphragm and related respiratory muscles.

## 5. Conclusion

Incremental load respiratory muscle training effectively improved the children's lung function over the long term, improved the strength of their inspiratory and expiratory muscles, and improved their quality of life.

## Figures and Tables

**Table 1:** Baseline characteristics at the time of study registration and severity grading evaluated using BSI scores.

Characteristics	Included subjects
Age (years)	11.05 ± 1.25
Gender (female/male)	9/6
Height (cm)	143.68 ± 5.69
Weight (kg)	36.00 ± 4.83
BMI (kg/m^2^)	16.68 ± 1.04
BSI severity classification	
Mild	3
Moderate	4
Severe	8

Statistically significant values are given in bold. BSI: Bronchiectasis Severity Index.

**Table 2:** Changes of expiratory muscles, expiratory exercise loading, expiratory work rate, and expiratory work, as well as the effect size of corresponding indicators.

Characteristics	Before training	12 weeks of training	24 weeks of training complete	*P* value1	*P* value2	*P* value3	Effect size
MEP (cm H_2_O)	63.28 ± 18.06	74.91 ± 18.48	90.87 ± 19.42	0.071	0.049	0.020	0.69
MEP %pred	50.49 ± 9.51	58.92 ± 10.27	71.59 ± 10.75	0.076	0.026	0.009	0.64
EEL (cm H_2_O)	34.17 ± 9.61	46.67 ± 10.46	71.67 ± 15.31	0.018	0.032	0.023	0.67
Expiratory work (J)	14.01 ± 4.56	23.91 ± 6.31	38.76 ± 11.57	0.116	0.092	0.04	0.38
Expiratory work rate (W)	17.45 ± 6.93	21.88 ± 7.00	27.57 ± 8.70	0.008	0.045	0.007	0.56

MEP: maximum expiratory pressure; EEL: expiratory exercise loading; *P* value1: comparison between before training and 12 weeks of training; *P* value2: comparison between 24 weeks of training and 12 weeks of training; *P* value3: comparison between after 24 weeks of training and before training.

**Table 3:** Changes in lung function-related indicators, LCQ scale score, and FSS score, as well as the effect size of corresponding indicators.

Characteristics	Before training	12 weeks of training	24 weeks of training complete	*P* value1	*P* value2	*P* value3	Effect size
FEV1 (liters)	1.72 ± 0.84	1.92 ± 0.94	2.08 ± 0.90	0.004	0.088	0.0004	0.67
FEV1%pred	67.99 ± 11.75	75.93 ± 11.79	83.65 ± 13.32	0.0004	0.035	0.0003	0.72
FVC (liters)	2.31 ± 1.01	2.58 ± 1.19	2.80 ± 1.15	0.011	0.015	0.001	0.68
FVC%pred	78.24 ± 10.98	86.17 ± 12.89	94.69 ± 13.12	0.001	0.005	0.0001	0.79
FEV1/FVC%	83.67 ± 9.40	85.93 ± 12.40	87.42 ± 11.74	0.35	0.4	0.173	0.13
PEF (L/s)	3.94 ± 1.94	4.61 ± 2.18	4.98 ± 2.27	0.001	0.02	0.0001	0.79
PEF%pred	67.51 ± 9.79	79.53 ± 12.73	86.56 ± 9.45	0.001	0.04	0.0001	0.80
FEF25 (L/s)	3.08 ± 0.60	3.49 ± 0.61	3.98 ± 0.61	0.005	0.015	0.0001	0.77
FEF25%pred	56.54 ± 6.08	65.18 ± 6.10	72.45 ± 6.47	0.071	0.723	0.007	0.37
FEF50 (L/s)	1.55 ± 1.22	1.75 ± 1.38	1.97 ± 1.41	0.118	0.006	0.001	0.69
FEF50%pred	45.82 ± 20.51	51.60 ± 21.24	59.54 ± 23.02	0.042	0.019	0.0003	0.75
FEF75 (L/s)	0.59 ± 0.41	0.71 ± 0.60	0.95 ± 0.76	0.409	0.007	0.045	0.50
FEF75%pred	33.24 ± 15.65	39.13 ± 21.81	51.24 ± 30.15	0.372	0.04	0.042	0.52
MMEF75/25 (L/s)	1.27 ± 1.03	1.46 ± 1.27	1.71 ± 1.34	0.303	0.009	0.012	0.58
MMEF75/25%pred	42.01 ± 18.74	47.81 ± 20.42	57.61 ± 23.01	0.109	0.018	0.001	0.72
FSS score	52.25 ± 1.09	40.08 ± 0.72	29.67 ± 0.68	0.0003	0.0003	0.0003	0.98
LCQ							
Physical	2.87 ± 036	4.22 ± 0.24	5.50 ± 0.13	0.0001	0.0001	0.0001	0.88
Psychological	3.56 ± 0.42	4.56 ± 0.21	5.66 ± 0.14	0.007	0.001	0.002	0.67
Social	3.63 ± 0.47	4.52 ± 0.31	5.73 ± 0.15	0.006	0.002	0.002	0.66
Total (3–21)	10.05 ± 1.16	13.30 ± 0.72	16.89 ± 0.32	0.0001	0.0001	0.0001	0.78

*P* value1: comparison between before training and 12 weeks of training; *P* value2: comparison between 24 weeks of training and 12 weeks of training; *P* value3: comparison between after 24 weeks of training and before training; FEV1: forced expiratory flow in 1 s; FVC: forced vital capacity; PEF: peak expiratory flow; FEF25–75%: forced expiratory flow 25–75%; MMEF75/25: maximal midexpiratory flow75/25; LCQ: Leicester Cough Questionnaire; FSS: Fatigue Severity Scale.

**Table 4:** Changes of inspiratory muscles, inspiratory exercise loading, inspiratory work rate, and inspiratory work, as well as the effect size of corresponding indicators.

Characteristics	Before training	12 weeks of training	24 weeks of training complete	*P* value1	*P* value2	*P* value3	Effect size
MIP (cm H_2_O)	59.37 ± 16.33	66.30 ± 17.94	80.42 ± 20.40	0.148	0.072	0.035	0.57
MIP %pred	55.99 ± 10.22	59.04 ± 11.62	68.93 ± 14.49	0.57	0.333	0.321	0.29
IEL (cm H_2_O)	35.83 ± 8.89	44.17 ± 10.52	59.17 ± 15.13	0.058	0.159	0.11	0.60
Inspiratory work (J)	12.92 ± 4.98	19.35 ± 4.21	30.96 ± 11.16	0.791	0.544	0.426	0.35
Inspiratory work rate (W)	9.77 ± 4.36	13.24 ± 4.45	17.10 ± 6.12	0.602	0.576	0.147	0.14

MIP: maximum inspiratory pressure; IEL: inspiratory exercise loading; *P* value1: comparison between before training and 12 weeks of training; *P* value2: comparison between 24 weeks of training and 12 weeks of training; *P* value3: comparison between after 24 weeks of training and before training.

## Data Availability

The data used to support the findings of this study are included within the article.

## Additional Points

*Clinical Messages*. A 24-week long-term respiratory muscle training can effectively improve the lung function and respiratory muscle strength in children with bronchiectasis. *Contribution of Paper*. Improvement of pulmonary function is very important for children with bronchiectasis. Respiratory muscle training can not only improve the pulmonary function of children with bronchiectasis but also continuously improve the respiratory muscle strength.
